# The Treatment of Diphtheria

**Published:** 1882-05

**Authors:** J. H. Carstens

**Affiliations:** Detroit, Mich., Professor of Therapeutics, Materia Medica, etc., Detroit Medical College


### Independent Practitioner.

VOL. III.——*May, 1882.*——NO. 5.

### ORIGINAL COMMUNICATIONS.

ARTICLE I.

### THE TREATMENT OF DIPHTHERIA.

BY J. H. CARSTENS, M.D., DETROIT, MICH.,

Professor of Therapeutics, Materia Medica, etc., Detroit Medical College.

When considering the treatment of diphtheria the question naturally arises, what is the pathology, etiology, etc? Our treatment will vary according to the answer we make. Some hold that it is primarily a constitutional disease, manifesting itself by a deposit on the pharynx, trachea, etc., while others claim that the exudation is the primary trouble, and the system is infected from this, at first, local trouble.

My views are different. I believe that both theories are true; that in some cases we have at first to deal with a local trouble, and in other cases that we have a constitutional disease which manifests itself by the characteristic membrane. In small-pox very different symptoms develop according to how the poison is introduced in the system; if by inoculation, the disease runs a mild course; while if by the air-passages or other mucous surfaces, severe symptoms result. Why should not the same be the case with diphtheria? If the poison is first deposited on the tonsils (as is often the case), and these spots be thoroughly destroyed with caustic early, but slight constitutional disturbances are observed; if, however, the poison enters the system (the blood) by other channels and is allowed to accumulate before the deposit takes place in the larynx or pharynx, so that we can make a diagnosis, we will find a very grave systemic affection.

Therefore I treat cases where only a deposit is found on the tonsils by the application of caustics, such as carbolic acid, and especially muriatic acid diluted—general use, one part acid and three parts honey or syrup. This is applied three or four times a day. When, however, the whole force of the disease is concentrated in the larynx we must use an atomizer or insufflator. In such cases we will often have trouble in making the parents understand the usefulness of caustics, in which some have great faith.

In all cases, however, the constitutional treatment should not be neglected, as it is of paramount importance, even in the mild form of the disease. Considering diphtheria as a zymotic disease and quinine as the best antizymotic we have at present at our command, I have more faith and place more reliance on the use of quinine than on any other remedy. I believe in using it freely. Get the system under the influence of the remedy as soon as possible, and when cinchonism is produced, lessen the dose, but continue to use it during the course of severer symptoms of the disease. Alcohol is also indicated as a general stimulant and tonic, and, on account of the adulterations of wines and brandies, I always rely on our own domestic whiskey. Diet of course should be easily digested and nutritious—beef tea, oysters, raw eggs, etc. The many other remedies which have from time to time been suggested I have nearly all tried, such as benzoate of soda, pilocarpine, etc. In some cases benefit seemed to be derived by the use of one or the other remedy, in others no effect could be observed, so that I have finally made up my mind that no remedy so far proposed has any specific effect in diphtheria.

In considering the treatment of the local manifestations of the disease, I believe that we should aid nature in her effort to throw off the false membranes. We find that the natural unaided course to get rid of the membranes is as follows : between the false and the mucous membranes pus is developed, and a secretion of mucus also aids to separate and assist in casting off the deposit. Therefore the best plan would be, and it really is, to aid the effort of nature, to produce a formation of pus and secretion of mucus.

To accomplish this HEAT AND MOISTURE will give the best results. Inhalation of steam with an atomizer I consider preferable. This should be done almost constantly or at least every half hour for ten or fifteen minutes at a time. Many efforts have been made to find some solvent for the diphtheritic membrane. A number of chemicals have been found to more or less perfectly dissolve the false membrane in a test-tube, but when applied to the throat of a patient the result is generally *nil.* These solvents also as a rule injure the mucous membrane, for if they do not they have no effect on the deposit. I have tried and seen tried nearly all these different preparations, such as lactic acid, lime-water, etc., but the issue was the same; some patients would get well and some die. We have no specific in this line. Astringents give no better result. I could never observe any good from their use.

The insufflation in powder form of remedies which destroy the bacteria, etc., such as sulphur, have been proposed. This plan I have also tried, using sulphur, alum, or bicarbonate of soda, but without any marked specific benefit.

The readers of THE INDEPENDENT PRACTITIONER must begin to think that I have but little faith in any remedy; and they are about right. I have no faith in any specific remedy, but believe that we should simply follow a general course of treatment. and also to fulfil indications as they arise.

Externally ice has been strongly urged, and is found to be good when much inflammation exists, although the application of heat gives relief in as many cases; counter-irritants are of no use, except perhaps mild preparations of iodine.

As a general rule I treat cases about as follows: get the system under the influence of quinine; give nutritious diet and stimulants—yes, give large doses of whiskey when prostration is extreme; if cerebral symptoms are marked give bromides and other nerve sedatives; use a steam atomizer with plain steam or with a little bicarbonate of soda, which I think aids in loosening the false membrane; sometimes I also use the old mixture of tincture of iron and chlorate of potassa, but prefer the following:

℞. Tr. Iodin. Co., ʒ ss.

Ammon. Chlorid., ʒ ss.

Glycerinæ, ℥ ss.

Aquæ, ℥ ss. M.

Teaspoonful every two hours.

The iodine acts as a disinfectant and removes to a great extent the disagreeable odor, but it also acts on the mucous membrane like the chloride of ammonium, which induces a secretion of mucus ; the glycerine when diluted with water has no effect, except to make the medicine pleasant to take. Externally I use sometimes ice, but as a general rule iodide of potassium or the compound iodine ointments. Finally, if the larynx fills up and danger of asphyxia exist, tracheotomy—with which, however, we should not wait until the patient is in *articulo mortis*, but perform the operation in time, if we can get the consent of the parents, or refuse to perform it. I had the misfortune to have a case die while performing the operation, and I know of a number of other cases like it. In some the question was, Did the child die of diphtheria or the anæsthetic ? Other physicians have asked the same question, and came to the same conclusion that I did—not to operate except without anæsthetics. In general, therefore, I might sum up, without offering any specific or anything new, about as follows :

1. Constitutional treatment by quinine, whiskey, nourishing food.
2. Locally, heat and moisture, by steam, with or without solvents, weak solution of iodine, etc.
3. Externally, mild counter-irritants such as compound iodine salve ; and, finally, tracheotomy performed without the use of an anæsthetic.

